# Realistic medicine to improve the quality of care in Scotland

**DOI:** 10.2471/BLT.17.030617

**Published:** 2017-06-01

## Abstract

Scotland is in the throes of a public health revolution, as it rolls out its new “realistic medicine” approach. Catherine Calderwood tells Fiona Fleck why empowering both patients and clinicians is improving the quality of health care.

Q: What attracted you to health policy-making?

A: While I was practising obstetrics in 2008–9, changes were made in antenatal screening and scanning policies. These were good policies, but developed by someone who didn’t understand the service very well. I saw a job advertisement in the *BMJ* for a senior medical advisor for women and children’s health, and thought: “That’s the person advising the government on these changes. I could do better”. I felt there was a disconnect between the policy-makers and the practitioners. I’d never been on a government committee before, but felt I was suited to the job as a translator or a bridge between the National Health Service (NHS), the service providers, the politicians and the government.

Q: What new initiatives have you brought in as Scotland’s CMO?

A: My main policy initiative was my first annual report on the health of the nation with data on smoking, alcohol, physical activity and other aspects of health. I had been hearing from my medical colleagues when going around the country that they were not comfortable with the way they were practising medicine. There was a lot of over-medicalization, they were stressed with an ever increasing workload and concerned about harm to patients and waste of resources. As part of that report, I wrote to doctors asking about their approach to decision-making and how they try to reduce harm and waste. I didn’t know what the response would be, as CMOs have not traditionally written to doctors to ask for their opinion or to start a discussion.

Q: What was the response?

A: I received hundreds of responses from doctors as well as nurses, midwives, occupational therapists and members of the public. They welcomed the chance to participate in decision-making and to discuss new approaches to personalized care. They pointed out the variation in practice, asking why one health-care intervention leads to better outcomes in one part of Scotland than the same intervention somewhere else in Scotland. I didn’t have the answers, but at least we have asked the question: “Why is there unwarranted practice variance?” and got professionals to look at their own data and practice. For example, in orthopaedic care in one unit 90% of patients get to the operating theatre for repair of a hip fracture within 36 hours, in another unit it is only 35%, but we know that a shorter time to operation gives the best outcome. I didn’t expect such a big response to our questions. The report has been shared almost 10 million times on Twitter and become something of a social movement in Scotland.

Q: What do you mean by “a social movement”?

A: One in which everyone feels they can participate in public health discussion and decision-making to bring about change. This new movement has allowed us to become involved in other health initiatives across the world such as Choosing Wisely in the United Kingdom and the United States of America, Slow Medicine in Italy and Prudent Health Care in Wales. In our second annual report released in February, we describe how we are trying to realize this vision of realistic medicine in Scotland and how we will be engaging with the public to achieve this. We also reported on how the concept of realistic medicine has been taken up by doctors and other health professionals. Now, on the policy side, we have policy colleagues who have “realistic medicine” in their job titles.

Q: What do you mean by “realistic medicine”?

A: The dictionary definitions of “realistic” are important here. “Having or showing a sensible and practical idea of what can be achieved or expected” and “representing things in a way that is accurate and true to life”. We would want all aspects of health and social care to be delivered in this way, and, in particular, delivering care in a way that is right for the person and their family receiving care and that takes into account the priorities of people receiving care.

“We are working together to make the consent process a more shared decision.”

Q: How are you incorporating a personalized approach into health-care delivery in Scotland?

A: One example is our review of the patient consent process. We are working together to make the consent process a more shared decision, by shifting to a request for treatment, rather than patients consenting to have something done to them. Patients and practitioners feel that they have permission to act differently. So, for example, just because interventions are available, it doesn’t mean practitioners must prescribe them every time prescription can be justified. Maybe we should see the doctor as an arbiter between several options, for whom no treatment is also an option. General practitioners, say: “We’ve been practising medicine in this way for years, now we have the green light to do this formally”.

Q: Are there other examples of how you are testing this approach with health-care professionals and the public?

A: We are working on a health literacy plan so that people have the relevant background knowledge to inform their discussions with health-care professionals. That also means helping people who may be struggling with decision-making regarding their health. In a survey of 1000 people from all walks of life and all parts of Scotland, we are going to ask: “What is realistic health?”, “What are your priorities for health and health care?” We will also get people’s views at face-to-face events, where we will be talking to members of the public about realistic medicine. Also, we are organizing citizens’ jury events, where the pros and cons of a selected health topic are debated. The professional view has been that we need to change our practice, but that’s no good if we haven’t engaged with the public.

Q: How are you working with practitioners in Scotland to improve the quality of health-care delivery?

A: We’re reviewing practice in our ophthalmology services and how we can treat people at home, rather than sending them into hospital care. This work grew out of the realization of the regional variation in these services that I mentioned already. The other area we are looking at is unnecessary follow-up of outpatients. For example, instead of insisting on a fixed return visit after six months for all patients, we are allowing people easier access to a follow-up visit if they have a problem. We started this in gastroenterology and dermatology, areas of high patient volume, a year ago. It’s early days yet, but we have already reduced the number of unnecessary appointments and achieved a more streamlined service for the people who need to be seen. For example, we have integrated health and social care. This allows services that were previously run separately to understand their individual roles better. 

Q: How are you trying to reduce harm caused by over-diagnosis and over-investigation in health care?

A: We developed a list of five questions that people are encouraged to ask their doctor or other health-care professional. The idea is to empower them to have a conversation, rather than accepting a doctor-knows-best approach. The questions are: “Is this test or treatment really necessary?” “What are the benefits?” “What is the downside?” “What are the side-effects?” and “What happens if we do nothing?” One of our health boards started piloting distribution of the questions in January, by posting them on the wall of every outpatient clinic. Their design team is also creating dialogue cards, the size of a credit card, with the questions on them to give to people being admitted for an operation. This can help reduce inhibitions about asking questions. Clinicians tell us that these questions allow them to explore all the options with patients, including doing nothing. When referred to a doctor, patients often think this means there must be an intervention and are often relieved when they don’t need an operation. 

Q: In which situations are patients opting for “no treatment”?

A: Physicians at haemodialysis clinics have started talking to the patients about the real consequences of treatment. They say: “You are eligible for treatment, but it means you will be in hospital for three days a week, and have regular tests and clinic visits. You can choose not to have haemodialysis: it may shorten your life, but you will have a better quality of life.” About 100 patients in Edinburgh have opted for no treatment.

Q: What are the challenges with offering the “no treatment” option?

A: Evaluation. “No treatment” and “treatment cessation” are not traditional outcome measures. We need to develop indicators for quality-of-life measures and patient-reported outcomes. Our patients are not interested in five-year survival, which is a current outcome measure for renal failure and cancer, but rather in shared decision-making and transparent conversations about what their treatment actually involves. Some of this discussion is inspired by research on what doctors would choose for themselves at the end of their lives. The evidence shows that 67% of doctors would not agree to being admitted to intensive care and 88% of doctors would not have haemodialysis at the end of life. The idea that as informed medical specialists we would choose one thing for ourselves but advise another for patients, made no sense to me. There is recent evidence that doctors overestimate the benefits of treatment and under-estimate the risks and side-effects. When the outcomes are not as good as we first thought, we tend to be reluctant to discontinue things that don’t work.

“The traditional way is ‘here is a leaflet’, but this is not the way people interact anymore.”

Q: How are you working with the public to improve the quality of health care?

A: Health literacy is very important, as well as public engagement. Shared decision-making won’t be right for everyone – some patients prefer to be guided by the doctor – but the fact is that we are having the conversation and giving people the chance to ask questions. Scotland has five medical schools and each plans to incorporate the principles of realistic medicine in their teaching, so the doctors of the future will be encouraged to share decisions with their patients. This reflects how society has changed. Today people get their information from diverse sources. The traditional way is “here is a leaflet”, but this is not the way people interact anymore. Doctors told us that they weren’t communicating enough with patients under time- and work-load pressure. I hope doctors now feel better able to regain their personal approach with patients, which they felt they had lost.

